# Atypical Manifestation of Disseminated Gastrointestinal Kaposi Sarcoma in a Newly Diagnosed HIV Patient: A Case Report

**DOI:** 10.1155/crgm/6919033

**Published:** 2025-10-05

**Authors:** Masood Muhammad Karim, Shazaf Masood Sidhu, Abdul Hadi Shahid, Asad Diwan Muhammad, Abdullah Bin Khalid

**Affiliations:** ^1^Section of Gastroenterology, Department of Medicine, Aga Khan University, Karachi 74800, Pakistan; ^2^Department of Oncology, Fauji Foundation Hospital, Rawalpindi, Pakistan; ^3^Medical College, Aga Khan University, Karachi 74800, Pakistan; ^4^Section of Histopathology, Department of Pathology and Laboratory Medicine, Aga Khan University, Karachi 74800, Pakistan

**Keywords:** acquired immunodeficiency syndrome, dysphagia, HIV, human Herpesvirus 8, Kaposi sarcoma

## Abstract

**Background:**

Kaposi sarcoma (KS) is a multifocal angioproliferative disorder linked to Human Herpesvirus-8 (HHV-8), presenting in four forms: classic, endemic, iatrogenic, and AIDS-related. AIDS-related KS remains prevalent among HIV-positive individuals despite widespread use of antiretroviral therapy. Gastrointestinal (GI) involvement is the most common extracutaneous manifestation, primarily affecting the upper GI tract. It is often asymptomatic and typically requires endoscopic evaluation. While commonly associated with advanced HIV, GI KS can occasionally precede an HIV diagnosis. Despite its strong link to HHV-8, disseminated KS may develop in HHV-8–negative patients with late HIV diagnoses, raising concerns about AIDS progression. Management primarily includes highly active antiretroviral therapy (HAART), while liposomal doxorubicin is reserved for extensive disease to ensure symptom control, lesion regression, and improved survival.

**Case Presentation:**

This case report describes a rare presentation of disseminated GI KS in an HHV-8–negative patient newly diagnosed with HIV, indicating potential AIDS progression. The patient exhibited violaceous cutaneous lesions and GI symptoms, including abdominal pain and dysphagia. Endoscopy revealed esophageal masses and ulcer-like lesions in the stomach and duodenum, with histology and IHC confirming KS despite negative HHV-8 status. Management included HAART and liposomal doxorubicin, with close IRIS monitoring. The patient remained hemodynamically stable and was discharged on HAART, chemotherapy, and prophylactic antifungal and antibiotic therapies following negative cultures and stable clinical status.

**Conclusion:**

This case highlights the need for vigilance in the atypical manifestation of HHV-8–negative disseminated GI KS in an undiagnosed HIV-positive patient, emphasizing the clinical presentation, diagnostic approach, and disease management. The patient's stabilization underscores the importance of early recognition, comprehensive diagnostic evaluation, and a multidisciplinary approach in managing complex cases.

## 1. Introduction

Kaposi sarcoma (KS) is a multifocal angioproliferative disorder first described by Moritz Kaposi in 1872. It is etiologically linked to human Herpesvirus-8 (HHV-8), also known as Kaposi sarcoma–associated herpesvirus. KS manifests in four distinct clinical-epidemiological forms: classic, endemic (African), iatrogenic, and AIDS-related [1]. Classic KS predominantly affects elderly men of Mediterranean or Eastern European descent. Endemic KS occurs in sub-Saharan Africa, independent of HIV infection. Iatrogenic KS is associated with immunosuppressive therapy, particularly in organ transplant recipients [2]. AIDS-related KS remains one of the most common malignancies in HIV-positive individuals, despite the advent of antiretroviral therapy [1, 2].

Almost half of KS patients with cutaneous lesions develop visceral manifestations, with the gastrointestinal (GI) tract being the most common site of extracutaneous involvement [3]. These KS lesions are mostly found in the upper GI tract, accounting for approximately 12% to 24% [4]. Although patients with GI lesions may present with abdominal pain, nausea, vomiting, malabsorption, or diarrhea, they are usually asymptomatic and may also manifest in an atypical manner [5, 6]. Therefore, KS patients with esophageal symptoms, such as dysphagia, require a thorough diagnostic evaluation for GI lesions. The high prevalence of these GI lesions, combined with their asymptomatic nature and atypical presentation in KS patients, necessitates endoscopic evaluation by a gastroenterologist. Complications of GI KS can include bowel obstruction and perforation [1, 6]. Although GI KS is highly associated with advanced HIV infection, it can occur at any stage of HIV and may rarely manifest before an HIV diagnosis [6]. HIV-associated GI KS is typically reported in patients with positive HHV-8 status; however, a negative HHV-8 result does not exclude the diagnosis of GI KS when clinical and histological findings are suggestive [1, 2]. Therefore, endoscopy with biopsy is warranted for histological and immunohistochemical (IHC) testing to achieve a definitive diagnosis in symptomatic patients [6, 7]. IHC testing is recommended for all tissue sections exhibiting spindle cell morphology, using biomarkers such as CD31, CD34, ERG, and CD117 [3].

Despite its well-established association with HHV-8, disseminated KS can occasionally manifest with GI involvement in HHV-8 negative patients, accompanied by a concurrent late HIV diagnosis, thereby increasing suspicion of AIDS progression [2, 8]. Management in such cases is primarily palliative, focusing on alleviating symptoms and preventing disease progression [9]. Treatment typically includes highly active antiretroviral therapy (HAART), tailored to the severity of HIV infection, and the burden of KS manifestations. HAART helps prevent new lesions, promotes regression of existing lesions, and improves survival [1]. In cases of disseminated and extensive disease, HAART could be used in combination with chemotherapy. Liposomal doxorubicin is considered the first-choice chemotherapeutic agent due to its preferential accumulation in KS lesions, offering a safe drug profile with a favorable response rate of approximately 72% [6].

This case report discusses a rare instance of an atypical esophageal manifestation of disseminated GI KS in an HHV-8–negative patient with a simultaneous HIV diagnosis, raising concerns about AIDS progression. It underscores the need for vigilance and thorough investigation in atypical presentations of the disease. The report aims to contribute to the timely evaluation and intervention required for managing AIDS progression.

## 2. Case Presentation

**Figure 1:**
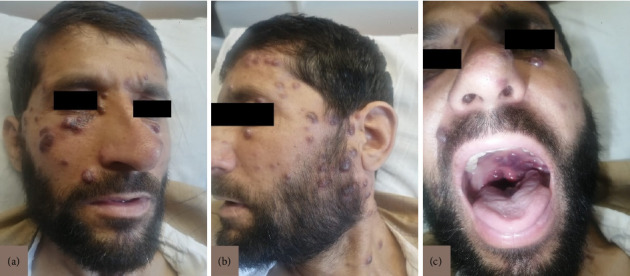
Clinical images of the patient showing multiple violaceous plaques and nodules, primarily involving the face and extending to the mucosal surfaces, consistent with KS. (a) Frontal view demonstrating extensive cutaneous lesions on the face. (b) Lateral view highlighting nodular involvement along the jawline, neck, and periauricular region. (c) Intraoral examination revealing mucosal involvement with characteristic violaceous lesions.

A 43-year-old male presented with a complaint of dysphagia for 3-4 months, along with epigastric pain for 1 month. Other associated symptoms included a productive cough and shortness of breath. On physical examination, the patient was alert and oriented, with vital signs within normal limits and bilateral coarse crackles upon chest auscultation. Skin examination showed multiple violaceous plaques and nodules, primarily on the face, extremities, and mucosal surfaces, findings consistent with KS cutaneous lesions (Figure 1).

**Figure 2:**
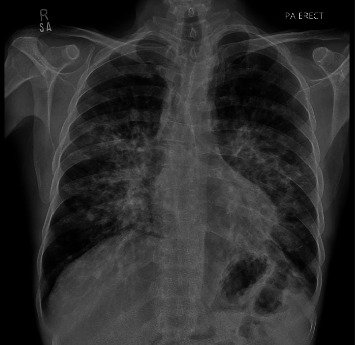
Chest X-ray (PA erect) showing bilateral perihilar nodularity, more pronounced in the infrahilar region. Given the history of Kaposi sarcoma, findings are suggestive of metastatic deposits, with atypical bacterial infection as a differential consideration.

Initial baseline laboratory investigations showed a normal coagulation profile, LFTs, electrolytes, and negative results for SARS-CoV-2. CBC revealed: Hb, 8.4 g/dL; HCT, 24.2%; RBCs, 2.82 million/μL; WBCs, 4.2 thousand/μL; PLTs, 223 thousand/μL. A chest X-ray revealed bilateral perihilar nodularity, more pronounced in the infrahilar region, suggestive of metastatic deposits (Figure 2). Given the history of KS, a whole-body PET-CT scan was performed, showing increased uptake in the nasopharynx, tonsils, base of the tongue, and widespread lymphadenopathy above and below the diaphragm. Further investigation revealed HIV positivity, with HIV-EIA and HIV-1 tests showing reactive results. General surgery was consulted, and an excisional biopsy of the right inguinal lymph node was performed. Histopathology showed spindle cells infiltrating and dissecting dermal collagen, with extravasated RBCs, extracellular eosinophilic globules, and lymphoplasmacytic infiltrates in the background, findings consistent with metastatic KS. IHC demonstrated positive staining for CD34, ERG, and CD31 but was negative for HHV-8. Consequently, a diagnosis of HIV and metastatic KS was established, and the patient was started on HAART. Upon HIV diagnosis, the patient's CD4 count was 295 cells/mm^3^. Given the presence of KS and suspicion of an opportunistic infection, the diagnosis was consistent with the progression to AIDS.

**Figure 3:**
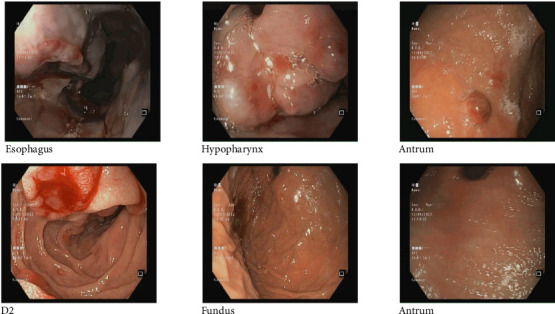
Upper GI endoscopy showing a nodular mass in the hypopharynx and multiple nodular lesions in the esophagus (21–27 cm) causing mild luminal narrowing. The gastroesophageal junction (38 cm) was examined, revealing gastric ulcers with indurated margins in the body and antrum, a maculopapular lesion in the antrum, followed by a volcano-like nodular lesion in the distal duodenum. Multiple biopsies were taken from these sites.

**Figure 4:**
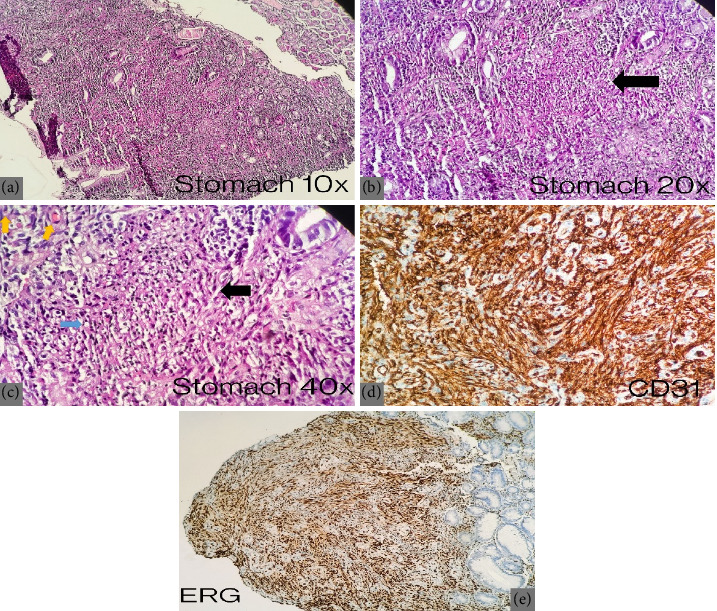
Microscopic examination of gastric biopsy sections. (a, b) Low-power views (10x and 20x) showing fragments of gastric mucosa with a spindle cell lesion in the lamina propria and submucosa (black arrow). (c) High-power view (40x) highlighting lesional cells with a fascicular growth pattern, elongated hyperchromatic to vesicular nuclei, eosinophilic cytoplasm, and indistinct cell borders (black arrow). The background reveals inflammatory cells (blue arrow) and numerous extravasated red blood cells (golden arrow). (d, e) Immunohistochemical staining for CD31 and ERG shows a positive pattern, while HHV-8 staining (not shown) is negative.

Additionally, the gastroenterology team was consulted for the evaluation of chronic dysphagia, and an upper GI endoscopy was performed during the same hospital admission based on clinical indication and the newly established diagnoses of HIV and metastatic KS. On esophagogastroduodenoscopy (EGD), a nodular mass was observed in the hypopharynx, along with multiple nodular masses in the esophagus extending from 21 to 27 cm, causing slight luminal narrowing. The scope was negotiated through with slight resistance, and multiple biopsies were taken. The gastroesophageal junction was examined at 38 cm, revealing a few ulcers with indurated margins in the body and antrum of the stomach, a maculopapular lesion in the antrum, and involvement of the distal part of the duodenum with a volcano-like nodular lesion (Figure 3). Multiple biopsies were taken from these sites. Histopathological examination of biopsy sections from the esophagus, stomach, and duodenum revealed a neoplastic spindle cell lesion with a fascicular growth pattern, displaying hyperchromatic to vesicular nuclei, eosinophilic cytoplasm, and indistinct cell borders. Inflammatory cells and numerous extravasated RBCs were noted in the background of the stomach and duodenal tissue. These findings are consistent with KS. IHC showed positivity for CD31 and ERG markers, negativity for cytokeratin AE1/AE3, and HHV-8 (Figure 4).

Several specialist consultations were obtained to provide comprehensive care. Pulmonology managed the diagnostic evaluation of the patient's respiratory symptoms, including the bronchoalveolar lavage (BAL) procedure. Oncology advised on the management of metastatic KS and supported the continuation of HAART. Infectious disease reviewed and confirmed the continuation of the patient's HAART, along with antifungal and antibiotic prophylaxis. Given the chronic cough and recent onset of fever, an infectious workup was initiated to rule out opportunistic infections commonly seen in HIV patients. The patient underwent an acid-fast bacilli (AFB) smear and GeneXpert test to assess for tuberculosis (TB). Both tests returned negative, ruling out TB. To further investigate the cause of his respiratory symptoms, a BAL was performed. Samples obtained were cultured for bacterial, fungal, and mycobacterial pathogens. All cultures, including the AFB smear, and fungal cultures for *Pneumocystis jirovecii* pneumonia (PCP) and Nocardia were negative. No pathogenic organisms were identified in the BAL samples, suggesting the absence of an active pulmonary infection.

During hospitalization, the patient's HAART regimen was continued alongside pegylated liposomal doxorubicin to maintain HIV viral load suppression and control KS lesions, with vigilant monitoring for immune reconstitution inflammatory syndrome (IRIS). Additionally, prophylactic antifungal and antibiotic therapies were maintained to prevent opportunistic infections, along with proton pump inhibitors to treat ulcer lesions in the GI tract. Throughout hospitalization, the patient remained hemodynamically stable. Given the absence of active infection, as indicated by negative cultures, and his stable clinical status, the patient was discharged with instructions to continue his HAART and chemotherapy cycles, along with the prescribed prophylactic antifungal and antibiotic therapies.

## 3. Discussion

KS is a vascular neoplasm primarily driven by infection with HHV-8, which preferentially affects individuals with compromised immune system [3]. KS lesions arise from dysregulated angiogenesis, driven by the combined effects of HHV-8 and cytokine-mediated pathways, resulting in spindle cell proliferation [10]. AIDS-related KS remains a significant opportunistic malignancy among HIV-infected individuals, even in the era of widespread HAART [6]. Despite the decline in incidence following the introduction of HAART, KS persists as a critical diagnostic and management concern, particularly in regions with limited healthcare access [11]. The clinical heterogeneity of KS can complicate diagnosis, especially in atypical presentations or when concurrent opportunistic infections obscure the findings. Skin biopsy typically reveals the characteristic proliferation of spindle cells, slit-like vascular spaces, and extravasated erythrocytes [9].

In this case, the patient presented with typical cutaneous lesions of KS, characterized by violaceous plaques and nodules, primarily on the extremities and mucosal surfaces. Additional systemic involvement was considered due to the patient's newly diagnosed immunocompromised status. GI involvement is the most common visceral manifestation, reported in 40% of KS patients [3, 9]. However, it often remains asymptomatic and undiagnosed. When symptomatic, patients may present with abdominal pain, nausea, vomiting, or GI bleeding. In our case, the patient only presented with abdominal pain and dysphagia, indicating esophageal involvement in addition to lesions in the stomach and duodenum [6, 12]. Esophageal manifestations, such as dysphagia, are uncommon presentations of KS and therefore require proper tissue evaluation to establish a definitive diagnosis [13].

In HIV-positive patients, KS with cutaneous lesions is highly associated with the development of upper GI involvement, reported in 40%–51% of cases [3]. Although KS diagnosis is frequently reported in individuals with advanced HIV disease, it can present at any phase of HIV infection [14]. In our patient, GI KS manifested early in an atypical manner, concurrent with the diagnosis of HIV infection. A literature review revealed one patient with a late HIV diagnosis who subsequently developed atypical KS. Another case report described a patient with cutaneous KS manifestations progressing to establish an HIV diagnosis [8, 15]. In such late-diagnosed HIV patients with preceding GI KS manifestations, AIDS progression could be considered, given the 50% incidence of GI KS in AIDS patients. Additionally, a previous study reported that KS presentation led to an AIDS diagnosis [1]. Similarly, our patient's GI KS manifestation and suspected opportunistic infection in newly diagnosed HIV infection raised suspicion of AIDS progression. In HIV patients, HHV-8 infection is highly associated with KS development, with 95% of lesions found to be infected with it [1]. However, HHV-8–negative status does not rule out KS if histological, endoscopic, or clinical characteristics correlate well with KS findings, as shown in a previous study [2, 16]. Therefore, upper GI endoscopy and biopsy are considered standard diagnostic evaluations in such patients with potential multifocal lesions. In GI KS, intraluminal filling defects may occur, with multiple coalescent lesions appearing as nodules that can protrude as polyps or masses. These features distinguish GI KS from typical candidiasis, which presents with discrete small mucosal plaques, cobblestoning, and rough, thickened epithelial surfaces [17]. Endoscopic findings in GI KS can be categorized as maculopapular, polypoid/nodular, and volcano-like nodular lesions [7]. Although these lesions are easily detectable by gastroenterologists, they can sometimes manifest as ulcer-like lesions, as seen in our patient. In such cases, histological and IHC testing play a crucial role in confirming the KS diagnosis [6, 7]. Additionally, these GI lesions are often accompanied by violaceous plaques on the face, trunk, and extremities, as reported in our patient. Biopsy typically reveals spindle-shaped cells and neovascularization with small-vessel proliferation, confirming the KS diagnosis, as evident in our patient [9].

The prognosis for HIV-related KS varies depending on the extent of the disease and the effectiveness of HAART. The management of HIV-related KS involves addressing both the underlying HIV infection and the neoplasm itself [9]. Initiation or optimization of HAART is paramount to restoring immune function and achieving viral suppression. HAART alone can lead to significant regression of KS lesions, particularly in patients with early-stage disease or localized cutaneous disease. For more advanced or disseminated KS, additional therapies, including liposomal doxorubicin or paclitaxel, are considered [1, 6]. The choice of therapy was guided by the extent of the disease and the patient's overall performance status. Liposomal doxorubicin is preferred due to its efficacy and favorable toxicity profile [6]. In this case, timely diagnosis and aggressive management were crucial for achieving disease control and preventing AIDS progression. In our patient, HAART was initiated alongside systemic chemotherapy due to the extensive disease burden and rapid progression. Close monitoring for IRIS, which can paradoxically worsen KS symptoms following HAART initiation, was also undertaken.

This case reinforces the importance of maintaining a high index of suspicion for GI KS presenting atypically as dysphagia in newly diagnosed HIV patients with cutaneous KS lesions, despite negative HHV-8 status, even in the HAART era. Timely diagnosis and management are essential to prevent AIDS progression. Further research into immune-modulating therapies and the role of checkpoint inhibitors in KS treatment holds promise. This case contributes to the ongoing discourse on KS by illustrating the interplay between HIV management and GI malignancy control.

## 4. Conclusion

This case highlights the complexity of managing a newly diagnosed HIV patient with suspected opportunistic infection and dysphagia due to atypical GI KS malignancy. Despite negative results for TB, HHV-8, and other opportunistic infections, the persistence of GI KS necessitated a thorough diagnostic workup and a comprehensive, multidisciplinary approach incorporating antiretroviral therapy, chemotherapy, and supportive care to prevent AIDS progression. The successful stabilization and optimization of the patient's management plan underscore the importance of individualized, patient-centered strategies in improving outcomes. Future efforts to enhance early detection, ensure equitable access to HAART, and explore novel therapeutic approaches for GI KS will be essential in reducing the morbidity and mortality associated with this condition.

## Data Availability

All data generated or analyzed during this study are included in this article. No additional data are available.

## Nomenclature

KS: Kaposi sarcoma
GI: Gastrointestinal
HIV: Human immunodeficiency virus
HHV-8: Human Herpesvirus 8
AIDS: Acquired immunodeficiency syndrome
HAART: Highly active antiretroviral therapy
IHC: Immunohistochemical
LFTs: Liver function tests
SARS-CoV-2: Severe acute respiratory syndrome Coronavirus 2
CBC: Complete blood count
Hb: Hemoglobin
Hct: Hematocrit test
RBC: Red blood cell
WBCs: White blood cells
PLTs: Platelets
PET-CT: Positron emission tomography–computed tomography
HIV-EIA: HIV enzyme immunoassay
EGD: Esophagogastroduodenoscopy
BAL: Bronchoalveolar lavage
AFB: Acid-fast bacilli
TB: Tuberculosis
PCP: *Pneumocystis jirovecii* pneumonia
IRIS: Immune reconstitution inflammatory syndrome
